# Impacts of attacks to female health care workers in three territories of Colombia

**DOI:** 10.1186/s13031-024-00582-9

**Published:** 2024-04-03

**Authors:** María Esperanza Echeverry-López, Alejandra Marín-Uribe, Isabel C. Garcés-Palacio, Yadira Borrero-Ramírez, Dora María Hernández-Holguin, Carlos Iván Pacheco-Sánchez, Rohini J. Haar

**Affiliations:** 1 Health Management and Policies Research Group, School of Public Health, Universidad de Antioquia UdeA, Medellín, Colombia; 2 Epidemiology Group, School of Public Health, Universidad de Antioquia UdeA, Medellín, Colombia; 3 Mental Health Research Group, School of Public Health, Universidad de Antioquia UdeA, Medellín, Colombia; 4 Health Policy Research Group, Department of Sociology, Universidad Nacional de Colombia –Sede Bogotá, Bogotá, Colombia; 5 Berkeley. School of Public Health, Division of Epidemiology, University of California, Berkeley, CA USA

**Keywords:** Medical mission, Armed conflict, International humanitarian law, Colombia, Women, Health impacts, Resistance, Attacks on health, War, Health workforce

## Abstract

**Background:**

This study explores the impacts of attacks perpetrated in the context of armed conflict, to female health workers in three Colombian territories.

**Methods:**

We conducted a document review of the reports and databases of the Colombian Truth Commission, 17 in-depth semi-structured interviews with experts on the national and regional armed conflict and the medical mission, and 26 female health workers who were victims of attacks.

**Results:**

Experts and female health workers reported attacks to health activities, facilities, equipment, and personnel, including attacks to traditional doctors belonging to indigenous communities. The most frequent attacks were threats and retention of health personnel; theft of supplies and medicines; damage and use of infrastructure and means of transport for purposes other than health care; and hinderance of health service provision. The attacks occurred in a framework of structural violence that intersects with poverty, racism, and gender bias. The impacts of these attacks include gender-based violence, significant disruption of the lives of health workers, and physical, emotional, psychological, social, and economic effects on the victims and their families. The government response to protect victims and populations has been absent or insufficient.

**Conclusions:**

Attacks to health care were reported in all the studied territories obstructing adequate health care. Impacts of these attacks affect negatively the professional and personal life of the workers and are aggravated by structural violence and absent or little institutional response.

**Supplementary Information:**

The online version contains supplementary material available at 10.1186/s13031-024-00582-9.

## Introduction

Colombia has experienced a six decade-long internal armed conflict. Since its inception in the mid-twentieth century, the conflict has left more than 9,395,274 registered victims [1]. Most of the victims of the armed conflict have been civilians. The main victimizing acts as of November 2022 have been forced displacement (8,366,104), homicide (1,085,339), threats (588,596), forced disappearance (190,709), loss of movable and immovable property (124,782), and violations of sexual integrity (36,572), especially against women from rural areas [1]. Health personnel have also been impacted while performing their professional duties with documented cases of physical damage, threats, kidnapping, forced disappearances, assassinations, sexual violence, and interference with the provision of care, such as theft of medicines and supplies, detention of ambulances to assassinate patients or for use in activities of armed groups, destruction of health posts and hospitals or the use of these for criminal activities [2–8].

In 2012, the Ministry of Health and Social Protection of Colombia, in Resolution 4481, introduced the term *Misión Medica*(Medical Mission) which include a wide range of health activities, facilities, personnel (does not include traditional healers or midwives), and equipment to carry out health care duties adequately. The resolution developed regulations to protect the Medical Mission [9]. The resolution also distinguished between “incidents”, which are attacks unrelated to the conflict, usually perpetrated by patients or their families, and “infractions”, which are attacks perpetrated by armed actors in the context of armed conflict, potentially in violation of International Humanitarian Law. Since international regulations usually use the term attacks on health to refer to infractions, we will use the term attack along the manuscript to refer to attacks perpetrated by armed actors. Based on the Geneva Conventions of 1949, its additional protocols of 1977 and International Humanitarian Law (IHL), the Colombian Ministry of Health and Social Protection defined five types of attacks: i) against life and integrity (threats, murders, forced disappearances, forced displacements, sexual violence); ii) against the infrastructure (use of health facilities for instance as trenches, centers of torture, or war operations; destruction and damage to hospitals, posts and health centers due to bombing or mining; and use of nonconventional ammunition against health facilities); iii) against health activities (theft of medicines and supplies, control of sanitary actions, confinements, inappropriate use of sanitary transport); iv) acts of perfection with the intention of harming or attacking the adversary; and v) violations of patient-health worker confidentiality [9].

In Colombia, women represent the largest proportion of the healthcare workforce. One study estimated that 80% of health workers are women [10]. Rural and lower-paid health workers make up the bulk of this workforce in Colombia [11]. These workers are particularly vulnerable to violence and their impacts—they are often poor, exposed to the frontline of conflict, interact with countless patients (many of them from armed groups), and have intersecting experiences of gendered and racial marginalization, as many are Afro-Colombian women.

While there is growing information on the number of attacks, their characteristics, perpetrators, and victims, both globally and in Colombia, a deeper understanding of their impacts is limited, specifically among female health workers. Given their critical role in providing care and the scant evidence on what they face in conflict and how best to support them, this study aimed to investigate the impacts of attacks on health on the personal and professional lives of women health workers.

## Methods

### Study design

This is a qualitative study that included document review and interviews. For the first part of the study, we review reports of the Colombian Truth Commission [8] and a national database on attacks to the medical mission. For the second part, we conducted (1) 17 semi-structured interviews with key informants (health workers, health administrators and leaders) with expertise on the national and regional armed conflict, the medical mission and attacks on health-related issues in Colombia and (2) 26 in-depth life-history interviews with female health workers who were victims of attacks during the performance of their duties [12].

### Document review

The Colombian Truth Commission, created in 2017, is a State entity that seeks to clarify the patterns and explanatory causes of the internal armed conflict. The Commission has reports published between 2016 and 2022 on various aspects of the conflict. We selected those relevant to attacks on health and women. One of those reports, the national database on attacks to the medical mission, was prepared jointly with the School of Public Health of the University of Antioquia and covers attacks between 1958 and 2019. We collected information both on the scope and scale of attacks on health—the frequency, geographic distribution, and trends in timing of the characteristics of the attacks: the types of attacks, the immediate sequalae, the perpetrators and the victims. We categorized them according to the guidelines provided by the Colombian Resolution 4481 [9].

### Interviews

#### Setting

The interviews with key informants with expertise on armed conflict were conducted in person in Medellin and through video and mobile communications with informants located in Popayan, Cali, Bogotá, Cucuta and Quibdo. In-depth life-history interviews were conducted in person in the territories, except for one that was through video call.

To conduct the in-depth life-history interviews with female health workers, we selected three territories: Cauca, Chocó and the Catatumbo area in the department of Norte de Santander. These departments had decades of high conflict intensity, high rates of poverty [13] and the presence of ethnic communities (indigenous and Afro-Colombian populations) who suffer discrimination by race, social class, and often lower occupational hierarchy, characteristics on which the victimhood of attacks to the medical mission converge. Armed actors have been disputing the territory for the abundance of natural resources, mining, and illicit crops. These disputes have resulted in forced displacement, assassination of leaders and human rights defenders, and permanent displacement of and restrictions on the population. Despite this, these regions have received scant attention from the State in social policy and economic development.

#### Sample and selection of participants

To identify key experts on armed conflict, we utilized purposeful sampling and snowball sampling to recruit additional experts within the initial sample networks. We contacted national and conflict-affected region experts, those who had published on the topic or were otherwise known to work on attacks on health, conflict, or gender dimensions of health services in Colombia. For the in-depth life-history interviews, we recruited female health workers who currently worked or had previously worked in the selected territories, purposefully sampling for those who had experienced attacks on health while carrying their duties as health care workers.

#### Theoretical approaches

**Fig. 1:**
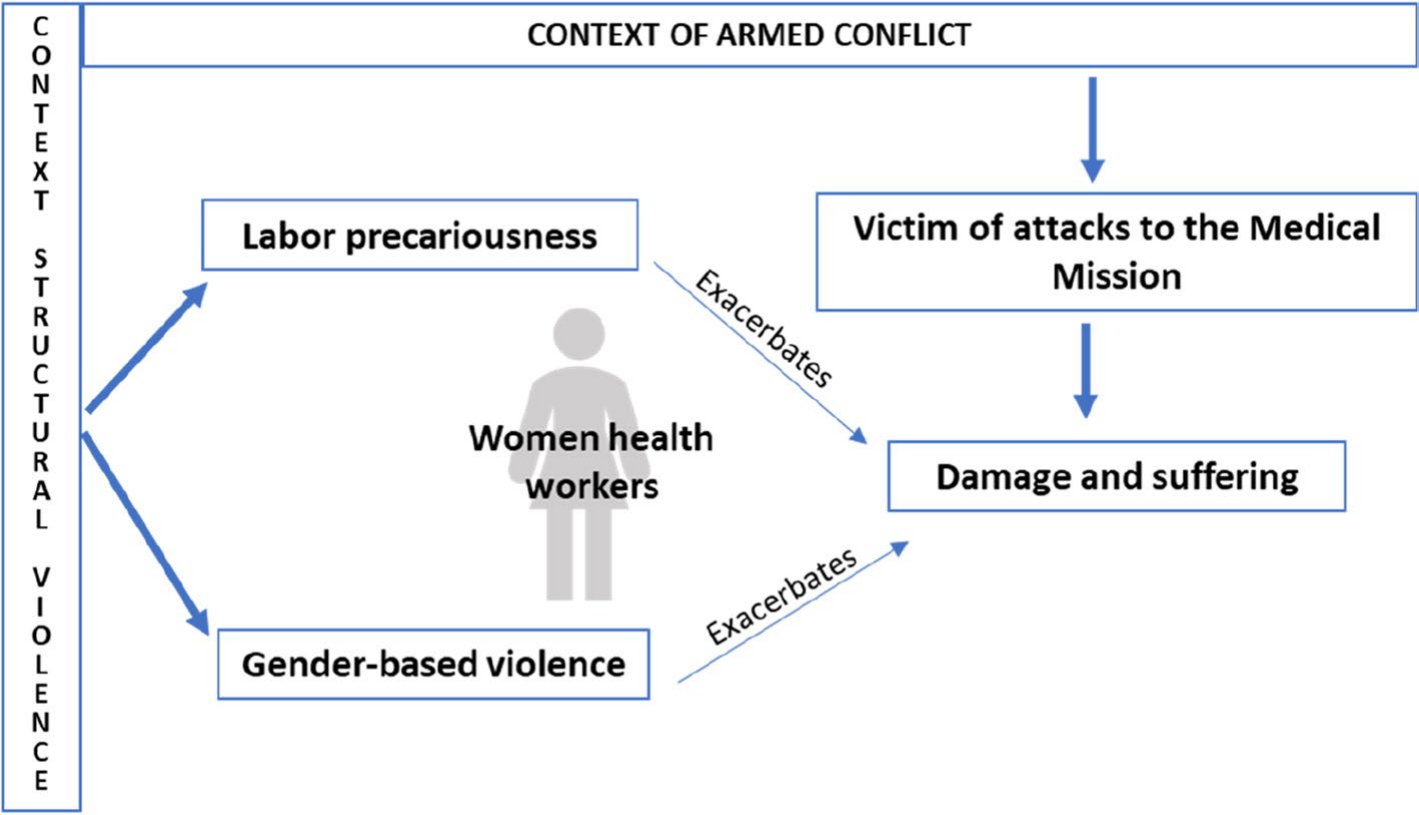
Conceptual framework

In this section we present a brief conceptual overview of the categories that informed the analysis of the study: the notion of victim as a starting point, a product of structural violence that materializes in job insecurity and gender violence that generate damage and cause suffering to women (Fig. 1). Together, these concepts underscore that understanding experiences of health attacks requires contextualization.

In Colombia, victimhood, product of the armed conflict, has been incorporated in the Victims Law 1448 of 2011 [14]. The concept of victim has moved from an individual perspective to a collective impact with a sociopolitical and cultural connotation. For this study, the victims were female health workers, their families, and communities in territories. However, women have not only been victims of the armed conflict, but, in their experiences, they have been victims throughout their lives of other forms of violence: structural violence and gender-based violence.

Structural violence, according to Galtung [15] is the product of deep asymmetries of power, class, gender, ethnicity, nationality, and territory, that put at risk basic life conditions. One way in which structural violence has materialized is through labor precariousness. For example, in the Colombian health sector it reflects in short-term work contracts, social benefits paid by the workers, layoffs, union dismantling, labor exploitation represented in low wages, and the lack of timely payment of salaries [16]. Labor precariousness was established from the globalization of markets since the late 1970s and in the Colombian health sector more systematically, with the implementation of the health reform of 1993 [17]. Furthermore, forms of gender discrimination in the labor markets aggravates labor precariousness: compared with men, women have lower-skill, poorly paid jobs, and long working hours that add to the burden of domestic care.

At the same time, forms of gender violence overlap in the lives of women. Gender-based violence (GBV) is also a form of structural violence, a product of power relations based on the differences that distinguish the sexes and that are integrated into the political and economic organization of our social world, constituting structuring processes that systematically perpetuate violence, causing damage and especially affecting to the poorest women [18]. In this research, GBV is a category that allows us to analyze the damages that women have experienced as a result of experiences of violence in their lives.

Finally, the conception of damage has moved, according to the National Center for Historical Memory (CNMH), from a causal and binary (good-bad, innocent-guilty) to a more complex and interdependent account of the experience of damage. It focuses on economic or material damage, physical disabilities, mental disorders, and impairment of collective legal rights such as the right to enjoy a healthy environment or the defense of cultural patrimony [19]. While in English, “impacts” holds similar meaning in terms of public health analysis, we utilize the more direct translation from Spanish, damages, to keep this paper consistent with legal and policy-oriented reporting in Colombia on this topic and to underscore that the impacts of attacks on health are overwhelmingly negative and need to be addressed.

### Data collection and analysis

We developed the interview questions based on the theoretical approaches described before. All interviews were conducted by members of the research team (female health worker victims of attacks were interviewed by female researchers). The interviews were conducted between February and April 2022. The duration of the interviews ranged between one and four hours. We ceased interviews when saturation of ideas was reached. Life-history interviews were halted before saturation was formally reached in the department of Chocó due to security issues in the region. The interviews were audio-recorded, transcribed, and deidentified. Subsequently, they were codified following preliminarily established categories from the theoretical approaches and literature review, with emerging themes being added inductively when relevant. We looked both for common patterns among the experiences and for disparate outlooks.

## Results

The results are presented in four parts: the first is an overview of the attacks, their characterization in the country's regulations and related trends between 1958 and 2019; the second is the profile of the participating women; the third is the types of attacks they faced, the damage caused by the conflict and the attacks; and finally, the social response.

### Magnitude of the attacks to the medical mission in Colombia: 1958–2019

**Fig. 2:**
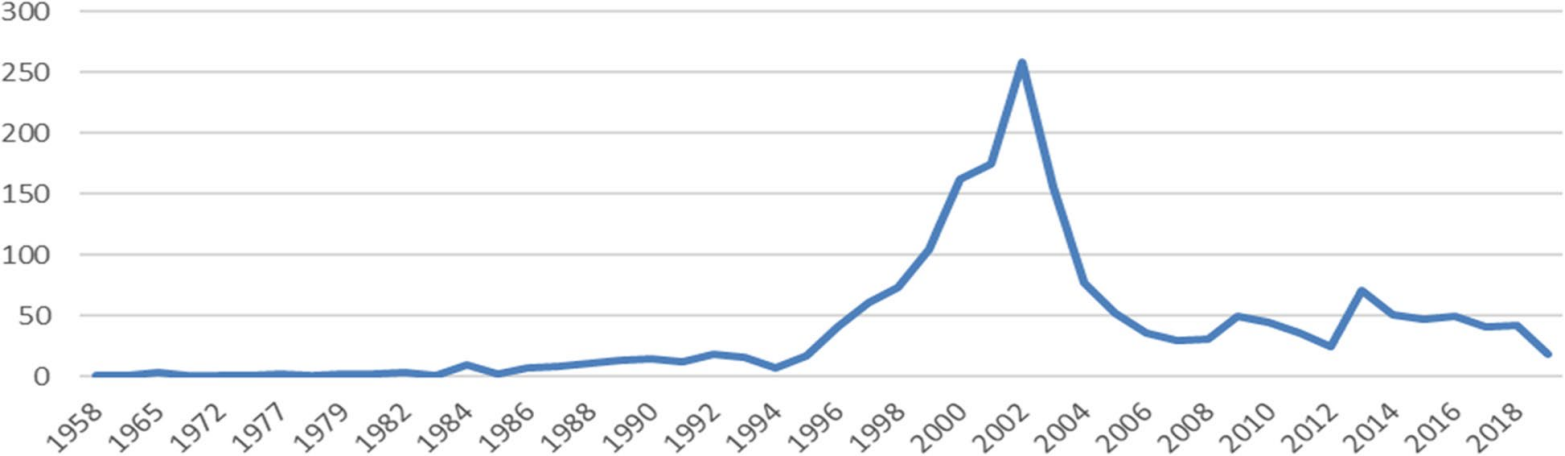
Incidents of attacks on health in Colombia, 1958–2019 Source: University of Antioquia and Truth Commission. Database Violations of the Medical Mission, Consultation date August 18, 2022

The Truth Commission documented the characteristics of health care attacks between 1958 y 2019, Table 1 shows a summary of the findings. According to the Commission, "in the last 30 years, there have been 565 homicides against the medical mission [personnel carrying out health care activities]" [8]. The year with the highest reported attacks was 2002, with a notable increase since 1998 and a stabilizing trend from 2008 onward (Fig. 2), a trend consistent with data on civilian violence in Colombia. As with the results of our interviews for this study, the Truth Commission found that the guerrillas were responsible for the majority of kidnappings and hostage-taking (74%), the paramilitaries for the largest proportion of homicides (39%), and unidentified actors for most threats (68%) [8].

**Table 1:** Characteristics of health care attacks in Colombia between 1958 and 2019

Health care attacks	Rural/Urban	Type of attack	Type of victim	Perpetrator
• Colombia 2124 individual victims 444 collective victims • Norte de Santander 165 individual victims 19 collective victims • Cauca 109 individual victims 46 collective victims • Chocó 61 individual victims 14 collective victims	Rural: 31.3% Urban: 27.5% Unknown:58.8%	• Against life and integrity: 72.5% • Against health infrastructure:14.3% • Against health activity: 12.7% • Violations of confidentiality: 0.4% • Acts of perfidy: 0.1%	• Health personnel: 93% including ancestral wise women and men and midwives • Patient companions: 5% • Civilian or combatant patients:1.4%	• Unknown: 39.8% • Guerrillas: 33.5% • Paramilitaries: 19.3% • State forces 3.2% • Actors acted jointly: 4.2%

Source: Comisión de la verdad. Caso «La salud entre fuegos» Infracciones contra la misión médica y la medicina tradicional, y violencia contra el sector salud en el conflicto armado colombiano (1958-2019). [Internet]. Informe Final - Comisión de la Verdad. 2022 [citado 12 de diciembre de 2022]. Available at: https://www.comisiondelaverdad.co/caso-mision-medica

The largest number of offenses occurred in the departments of Antioquia, Cauca, Nariño, Norte de Santander, Caquetá, and Arauca (map 1, Supplementary material). According to the number of inhabitants by department, the most affected were Arauca, Guaviare, Caquetá, Chocó and Vaupés (map 2, Supplementary material) [8]. In both cases, the departments selected for this research are among the most affected by attacks in Colombia. In all three, the most frequent attack was against life and integrity. The data show a change in the actors responsible for these events: during the period prior to 1995, the main culprits were the guerrillas; between 1995–2006, the paramilitaries; and from 2007 (coinciding with the partial disarmament of guerrillas and paramilitaries), the armed actors involved were not easily identified [20]. Our interview data corroborate this and mention that drug cartels were involved, but this could not be confirmed in official reports.

The attacks affected the populations that were left without health services and without health personnel. For example, in Cauca, there have been obstacles to the work of midwives due to restrictions on mobility and devastation of the territories that, in addition to environmental damage, destroy the medicinal plants that they and other ancestral wise women and men use in their health care practices [20].

The demobilization of armed actors, some paramilitaries in 2005, and FARC in 2016, decreased but did not eliminate the attacks. By October 2020, there were 242 attacks [21], and according to the Red Cross, between January 1 and May 5, 2021, there were 189 events that represented an increase of almost 400% compared to the same period in 2020, when there were 49 [22]. Of these, 126 occurred in social mobilizations (66.6%); and, in the areas of our interest**:** 29% were in Norte de Santander and 7% in Cauca.

### The voices of experts and victims: characteristics and experiences of attacks on health

#### Profile of participants

We interviewed 17 experts who were physicians, nurses, social sciences professionals, and social leaders working at NGOs, universities, Community Councils, and the Truth Commission of Colombia and had expertise, or experience with the broader issues around the conflict, the medical mission and/or attacks on health. Most of the experts were located in health-related institutions in major cities and towns, although in some cases, they provide extramural health care.

We also interviewed 26 women health workers, including 11 nursing auxiliaries, 7 professional nurses, 3 rural health promoters, 3 auxiliaries in different health areas, 1 ancestral wise woman and 1 ethno-health educator, who lived and worked in rural conflict-affected areas. The work of the rural health promoters is in the field and included monitoring the health situation of the assigned families and carrying out health promotion and home visits. Nine women have minority ethnic-racial identities: four identified as indigenous and five as Afro-Colombian. All other participants identified as mixed-race Colombian. Almost all the participants know the territory well because they were born and have lived or worked there during all their lives or a good part of it. Considering that the three territories have suffered from ongoing armed conflict for more than three decades, the participants have experienced long periods of victimization themselves within their families and communities.

We jointly analyzed the expert and female health worker interviews to synthesize the findings. We identified commonalities in the participants’ lives as well as the characteristics of violence against health that they faced.

#### Participants faced intersectional adversities within the conflict

The attacks reported by the participants share contextual elements: in addition to life stories traversed by poverty, their families and communities suffered victimizing events as a result of the conflict from an early age. These events include witnessing massacres, forced displacement, police repression, eviction of recovered lands or neighborhoods of invasion, forced recruitment, murder, threats or disappearance of coworkers, ancestral authorities and people from their territory. Armed actors threaten to harm their children if they deny health care or denounce the human rights violations perpetrated by them; they work in the country's health system in precarious conditions and, frequently, with long delays in payments. Some have a stable employment relationship and are part of social organizations and/or processes of struggle and resistance, and they have seen the intergenerational effects of the armed conflict.

Health workers living in territories with long-term armed conflict, such as those in this investigation, have faced discrimination, racism, and life trajectories crossed by gender-based violence such as sexual abuse and intrafamily violence. This illustrates that attacks are not episodic events but a continuum between the daily world and the work world.

#### Characteristics of attacks to the medical mission

Participants were victims of a range of different attacks on health. The most frequent included detention, threats, intimidation, and coercion to care for specific individuals. Infrastructure attacks included damage and improper use of health facilities, misuse of the medical emblem (i.e., for trafficking supplies), theft of medicines, supplies, and equipment for the care of combatants, and interference with triage decisions to prioritize armed actors.

*When we were [in the hospital], we were going to go into the operating room and some people [presumably members of the guerrilla bringing in a wounded combatant] got in from behind and pointed guns at us… the physician sutured him and they left the man intubated and threatened "if the man dies, you all die here."* (Health worker victim from Chocó)

In this testimony, the perpetrators shared the motives for their threats and violent actions to obtain information from the hospital:

*I went to sleep normally, and then I do not know what time it was, but I felt that someone was touching me…they put a knife to my throat and told me they were from a group…they told me I could not scream, I could not do anything...that they were going to enter the health center and that the order was to kill and rape all the women and men as well... and that they needed information from the hospital*. (Health worker victim from Catatumbo)

Table 2 summarizes these findings as well as the impacts and longer-term consequences of attacks reported by our participants. Guerrillas, paramilitaries, and police or military forces were reported to be responsible for most of the violence, although, coinciding with the quantitative data, in many cases, the identity of the responsible armed actors is unknown or imprecise.

**Table 2:** Violations of the medical mission against health workers in Cauca, Chocó and Catatumbo

Relevant attack experiences by participants	Other violent events experienced
**AGAINST LIFE AND INTEGRITY** -Retention -Kidnapping for care of armed actors in camps -Threats: if they report to authorities or if the combatant dies -Forced displacement -Sexual violence -Threats against family members if the demands are not fulfilled	- Confinement in Chocó and Catatumbo **In the three territories**: -Long periods of victimization due to long duration and continuity of the Colombian armed conflict -Kidnapping- Forced recruitment of children -Witnessing or learning of murders or disappearances of leaders, human rights defenders, or other health workers Violation of International Humanitarian Law, ignoring the condition of well-protected health units and medical transport -Destruction or damage to health units, leaving the population without services -Stigmatization: all the armed actors accuse health workers of being members of the opposite side for caring for combatants -Populations and health institutions without medicines or without care due to the resignation or displacement of health workers
**AGAINST INFRASTRUCTURE** - Armed actors camp in health institutions, use them as trenches, or to direct attacks against the enemy -Murder of patients transported in ambulances -Withholding "borrowing" medical transportation for purposes other than health -Bombing of health institutions	
**AGAINST SANITARY ACTIVITIES** - Impose or demand prioritization of care for wounded or sick armed actors - Armed combatants inside health institutions -Threat if the armed actor dies during the service -Prohibition of transit through the territory for rural health workers -Steal medicines or supplies from health units	
**VIOLATIONS OF CONFIDENTIALITY** -Forcing the disclosure of information about the injured or sick	

#### Sexual violence

Some participants described incidents of sexual or gender-based violence. One of the participants reported that she was sexually abused inside a health post by a man who identified himself as belonging to an illegal armed group. He questioned her about information from the hospital, threatened to sexual abuse and killed the staff at the health post. She was retraumatized by judgment from colleagues, by a State institution, and by the media. Today, she works in another municipality where no one knows about her case.

*The doctor from the medical mission and there later he was telling me that this had not been an abuse but an assault or something like that... they [mass media] said that the sexual abuse was from an ex-partner (...) is as if they took away from you the truth, they took away your power over your body, they took everything away from you... I would prefer that no one had known about this case.* (Health worker victim from Catatumbo)

It is likely that sexual violence suffered by health workers is underreported due to shame or fear of retaliation from the perpetrator or signaling from colleagues and the community.

### Damage from attacks on female healthcare workers in Chocó, Cauca and Catatumbo

Five types of damage emerged from the analysis: i) disruption of life projects, ii) emotional impacts, iii) collective impacts on the populations where the attacks occur, iv) moral damage represented in chronic suffering, and v) intergenerational effects.

#### Disruption of life project: family disintegration, material losses and stigmatization

Participants spoke of a life history traversed by the armed conflict that generated a range of complex ruptures from a peaceful life: forced displacement, and consequently impoverishment, as well as material losses of parents and family disintegration when the partner or children leave the territory (due to threats to health workers, or to avoid forced recruitment, or protect their lives). One participant described that, before the forced displacement, “*what I earned was enough for food and everything, we did not pay rent, the services there were very cheap and all that, everything was damaged.” (*Health worker victim from *Catatumbo)* Another noted that *“I felt helpless, so I told my husband: I'm going alone, we're going to leave the girls here, we're going to organize ourselves, you stay with the girls.”* (Health worker victim from Catatumbo)

One of the most frequent damages to health personnel, particularly women, has been stigmatization. All the armed actors – guerrillas, paramilitaries and public forces – have accused them of belonging to or collaborating with the group whose members they are treating or simply when they work in a territory where one of the armed actors is hegemonic. These damages transcend the attacks, and health care workers endure false accusations from the communities in which they work. [Health worker who witnesses the murder of her colleagues] *they threw us to the ground, they told us whatever they wanted, with our heads to the ground, arms behind, no one could speak, look, or move, some of the coworkers jumped into the water from the scare, they were pulled out with lead point, they were killed in front of us and accused us that we were paramilitaries, that for that reason they were going to kill us all*. (Health worker victim from Chocó).

*Where the army will find me! They said that I was a guerrilla, what had they not done to me, that was my fear, they kill me, rape me, what will they not do to me.* (Health worker victim from Cauca)

In some cases, especially when women belong to a health union, this has generated arbitrary arrests and criminal accusations that lead to the loss of labor rights and the restriction of participation and political rights. The testimony below reports the mental health impact of this kind of violence.

[Threat by an armed actor to a nurse] *Remember how trade unionists die, without eyes, without tongue, without hands, have a Merry Christmas. In addition, he gave me another pat on the shoulder and left. I felt like I was not coordinating my mind.* (Health worker victim from Catatumbo)

#### Emotional impacts: permanent damage to mental health and loss of autonomy

Attacks on health, especially cumulatively, resulted in chronic stress, depression, sadness, loss of freedom and autonomy when having to act out of fear. A pressing need for strength to move forward also emerges from the narratives as well as indifference and distrust between health workers. These impacts were often very long-term or permanent.

*It stayed with me forever, it was psychological damage because I cannot see weapons, they make me very nervous, too much, I get shaky, I cannot stand my jaws because of the nerves. Yes, more or less, that stayed with me forever*. (Health worker victim from Chocó)

These particularly gruesome and violent attacks left deep scars.

*Once the paramilitaries carry out a massacre, the people of the town say to me: nurse, you go and help collect the brains of those they killed because the dogs are eating them, psychologically, do you believe that one is not affected?* (Health worker victim from Catatumbo)

Victims also endured permanent disabilities, which impacted not only their livelihood but also their mental health. This is the case of a nurse who, at the age of 26, had one of her legs amputated due to a projectile fired by the guerrilla, which fell in the health unit in the middle of a confrontation with the military forces.

*I know that the emotional part, I am the one who has closed myself off, I have closed myself off a lot, a lot, so that they are going to see me, because they are very big consequences, not only the amputation, but it is also in the whole leg, a deformity, that another person sees me.* (Health worker victim from Cauca)

Cumulatively, health workers shared experiences of anxiety, depression and even suicidality.

*I never contemplated the possibility of suicide and at that time I was very depressed that I did contemplate suicide very often and I felt as if it were a cloak that wrapped me, but always, always dark.* (Health worker from Catatumbo)

In addition, living long periods of war in the territories where they perform their health care duties, has generated chronic suffering contributing to moral damages. For instance, the loss or absence of children because they are recruited or kidnapped by armed actors has created emotional wounds difficult to overcome.

#### Collective impacts: populations without health services and loss of ancestral knowledge

Collective impacts on the community include the destruction of health units due to bombings or other war actions and the hindrance or demotivation of the work of rural health promoters and ancestral wise women and men due to confinement or imposition derived from the territorial control of the armed actors. In these cases, populations are left without services, rural health promoters are afraid to work, cultural references and traditional knowledge in health are being lost, and the human right to health is restricted:

*Well, that* [the attacks] *affects a lot, because there are also some promoters who are there and who do not want to work, they go with that spirit and study, but when they see the tough situation with them, they surely listen to what they talk about, then they no longer want to work.* (Health worker victim from Cauca)

These impacts go on to increase sociomedical inequities in territories that, due to their precarious living conditions, require more State presence and health services.

*[There are too] many... pregnant girls because we could not get there with the planning programs, children without their vaccinations, even cases of tuberculosis arose out there... a woman died while having a baby, there was no promoter, there was no assistant, there was nothing.* (Health worker victim from Cauca)

#### Intergenerational effects: lives cut short and the pain of young people

*Intergenerational impacts were frequently shared by participants:* depression, silence, social isolation of the sons and daughters for living in the midst of the conflict, for the victimizing events suffered by their family or by their mother victim of attacks, or for separating from their parents who protect them by removing them from the territory. In contrast, there are the lives cut short of young people who join the ranks of armed actors for various reasons, but generally through forced recruitment.

*Psychologically, my God, it is hard to forget this* [intraurban forced displacement and violence in Chocó], *because even though years have passed, one always lives with that, with that fear and well, my son, the man, always maintains at home, very sad, I have asked him for psychological help, I have taken him to the psychiatrist, she sends him to do therapies and all that, because he is capable of staying in the room all day, without talking to anyone, even that depresses me, he gets depressed. And I always have that fear (…) And she* [the daughter] *is also very quiet, she is, quiet, alone”.* (Health worker victim from Chocó)

Despite everything, according to our interviews, women also display agency: coping and resistance practices are not addressed in this article, but it should be noted that they are active or positive coping strategies, refuge in religion, support from family and friends, the protection of sons and daughters by removing them from the territory, the change of workplace, and, to a lesser extent, the complaint to the authorities. In passive coping, "flight" emerges—for example, forced displacement—or the denial or minimization of the victimizing facts as a form of "behavioral disengagement". Among the resistance practices, although they are not the most frequent, it is worth mentioning the politicization of women, the participation or leadership in organizational processes, social and union struggles, and the "transmission" of that fighting spirit to their children and daughters.

## The Social response

The social response refers to the actions deployed by the State and by society, in this case, the communities affected by the damages of the attacks. Participants pointed to the feeble local government response to attacks and the lack of national or international action to protect health workers or hold perpetrators accountable (beyond medical mission advocacy). Participants narrate the fear of revenge and threats to the children or the family as obstacles to denunciation. There is also skepticism because government authorities do not meet their demands or do not take effective actions to protect them or the population. In some cases, officials and the public force are allied with armed actors; in others, they design judicial montages with false accusations to unjustly imprison the health workers and silence their voice, as a victim in Cauca narrated. It should be noted that in a few cases, the State has provided protection schemes, which are often viewed with mistrust by threatened health workers. For all these reasons, some women have quit their jobs or been forced to flee or move to other locations or experience the traumatic event alone.

*When the paramilitaries arrived, the threats to the coworkers from the municipalities began, then they began to displace people, threatened them and all that, and a very tough fight began with the departmental health directorate to get them to relocate the people (...) they did not relocated them [the personnel], that was a fight and they [paramilitaries] killed many, that is, the threats were carried out.* (Health worker victim from Cauca)

Colleague and community responses varied between silence and inaction resulting from fear and the need for self-protection and solidarity expressed in protection actions. In some cases, community members or leaders interceded to protect health personnel. Union membership was also noted, despite some stigmatization, to protect health workers within a close-knit group. In other cases, coworkers minimize the damage or show distrust toward the victim's traumatic experience, deepening the stigmatization. Normalization of violence or underreporting may also limit community engagement.

*(...) Some armed men came to her workplace [health post], it was, in the back of her home, her husband and children were there, they forced her, amid shouting, insults and threats to weapons, along with her husband to go out to the corregimiento park (...) finally the priest went out and interceded for [her] and her husband, he said that the only thing they did was work and serve the community, faced with this intercession, they let them go to his house at dawn.* (Health worker victim from Catatumbo)

## Discussion

Attacks on health have been reported since 1958 in Colombia and continue on to this day. The most frequent attacks were threats, homicides, the retention of health personnel to care for combatants, theft of medicines and supplies, and the use of health units and medical transportation for purposes other than health care. We found that the impacts transcended the attack episode itself. The psychological, physical, emotional, and economic damages disrupt the lives of health workers, families, and communities, remain in memory, fracture social cohesion, have intergenerational impacts and result in a loss of ancestral knowledge. These damages often generate intangible, enormous social suffering and impact not only the health system and health workers but also the community and even the environment and violate the right to health. Women health workers particularly experience the stigmatizing and isolating impacts of sexual violence and the challenges of intersectionality. Despite adversity, there are glimmers of hope that emerge from the research: women health workers face different strategies of family solidarity, refuge in spirituality, connections with their children, and social support. The participation of health workers in social struggles has also forged resistance practices.

In line with our results, Báez-Quintero [23] characterized attacks between 2002 and 2006, finding 128 cases with 194 attacks and 163 victims as follows: 29% doctors, 12% nurses, 10% protected persons, 9% drivers, 7% health promoters, 7% health assistants, and 11% unknown. It also reported victims from indigenous communities who were working on the transportation of equipment and the organization of health activities. Likewise, Garcés et al. [24] described the experience of the medical mission within the framework of the armed conflict in two Colombian municipalities and found violations against life and integrity – homicide, forced disappearance, personal injuries, threats, forced displacement, among others – as impacting the health infrastructure and violating professional secrecy.

Other research based on narratives from health care providers has found results similar to those of this study. In Colombia, Urrego [5] reported violence against the wounded and sick, health personnel, means of transportation and health infrastructure. They highlighted the participants’ limited knowledge of international human rights and the protection protocols of the medical mission. Additionally, Molano [25] documented the use of the wounded as part of strategic targets, of medical knowledge as part of the organization of combatants, and processes of militarization of civilian medical life, which, beyond being territorial control strategies, have threatened health care workers’ professional ethics as founded in our research.

Regarding the impact on women, other researchers have reported similar results. For instance, Marciales [13] stated that armed conflict results in differential effects on women. For example, the structural racism of Colombian society has deep impacts on women that increase violence and damage. Consistent with the aforementioned works, the present investigation provides a gender perspective with women in territories of long duration of the armed conflict, who from an intersectional approach are racialized (most of the impacted women are from racial and ethnic minorities), located in a medium or low position in the occupational hierarchy of the health sector in Colombia, experience socioeconomic disadvantages compared to other health professions, are mostly women heads of household, and assume double or triple workload for domestic and family care tasks. In this life situation, attacks on health deepen long-standing structural violence.

This research coincides with others that have raised the right to health in its components of availability, access, acceptability, and quality. Several international works have reported that armed conflicts decrease the use of health services such as prenatal care, documented in the conflicts in Nepal [26, 27]. Sri Lanka [28], Burundi and Uganda [29], and the availability of health services and health personnel due to the destruction of infrastructure or because health workers migrate or are killed in the context of these conflicts, as in the cases of Lebanon [30], Cambodia [31], Sri Lanka [28], and Syria [32].

In Colombia, a study by the National Institute of Health documented that between 1998 and 2015, maternal mortality was higher in municipalities with a high intensity of armed conflict and low birth weight of children, hypertension and cutaneous leishmaniasis were also more frequent [33]. Additionally, Garcés et al. [24] found that the conflict generated difficulties for the provision of and access to health services, including access to medicines, which is an attack to health care also reported in this study.

This study has important limitations. As reporting on attacks on health was captured from secondary sources, selection bias, as well as reporting bias, have likely impacted the findings: we suspect that attacks on health continue to be severely underreported and that some voices are more hidden than others. In our qualitative arm, decisions on who to interview could bias our findings, but we do not claim any representative results. Rather, this study explores important findings on attacks and their characteristics, as well as women’s perceptions of their impacts. We hope that follow-up studies can more systematically strengthen reporting on these incidents, access more data, and engage local voices.

This research team considers that mitigating the impact of violence against the medical mission requires long-term structural reforms because the impact of violence is linked to multiple factors within the persistent armed conflict. However, the protection of health personnel, ancestral wise men and women, ethnic authorities, and rural health workers; systematic and timely reporting and follow-up to attacks; and comprehensive care for victims are all actions that can be implemented or improved.

Based on the results, this research concludes i) the attacks to health care are a product of the internal armed conflict, that is aggravated in part by poverty, discrimination, racism, and gender violence; ii) that it is necessary, as proposed by the Colombian Truth Commission, to broaden the concept of attacks to include effects on traditional healers or midwives; iii) the damage from the attacks is aggravated by the job insecurity experienced by health workers and particularly economically disadvantaged women like those in this study. The way health institutions are organized limits institutional responses, such as the transfer of workers.

### Supplementary Information

**Supplementary Material 1.**
