# Supplementary material for: Impacts of attacks to female health care workers in three territories of Colombia
Source: Confl Health. 2024 Apr 3;18:25. doi: 10.1186/s13031-024-00582-9 (PMC10988842; doi:10.1186/s13031-024-00582-9)
Supplement: Supplementary file 1 — Supplementary Material 1. [file 13031_2024_582_MOESM1_ESM.docx]

**Map 1. Number of attacks on the medical mission by department**


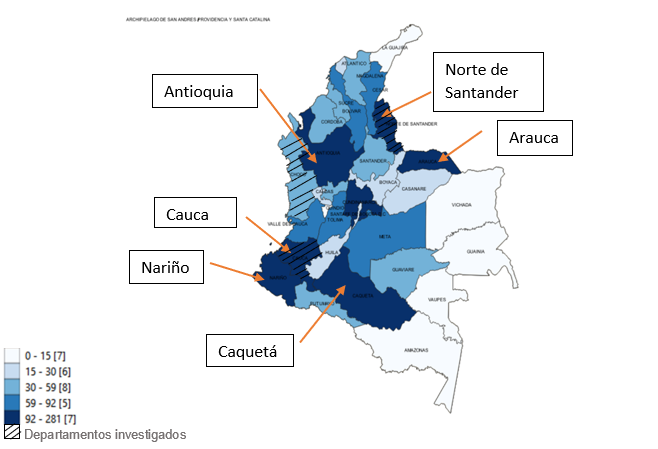


Source: University of Antioquia and Truth Commission. (2020) Database Violations of the Medical Mission. Consultation date August 18, 2022

**Map 2. Proportion of attacks to the medical mission per population by department**


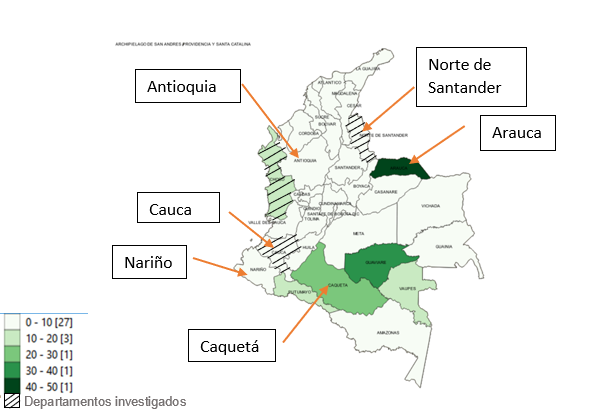


Source: University of Antioquia and Truth Commission. (2020) Database Violations of the Medical Mission. Consultation date August 18, 2022
